# Cedrol prevents UVB-induced photoaging by restoring mitochondrial function, metabolic homeostasis, and skin barrier integrity in HaCaT cells

**DOI:** 10.1080/13880209.2025.2583837

**Published:** 2025-11-10

**Authors:** Mo-Rong Xu, Chia-Hsin Lin, Hsun-Hua Lee, Sheng-Yang Wang

**Affiliations:** a Doctoral Program in Microbial Genomics, National Chung Hsing University and Academia Sinica, Taichung, Taiwan; b Department of Forestry, National Chung-Hsing University, Taichung, Taiwan; c Department of Chinese Pharmaceutical Science and Chinese Medicine Resources, China Medical University, Taichung, Taiwan; d Department of Neurology, Taipei Medical University Hospital, Taipei Medical University, Taipei, Taiwan; e Department of Neurology, School of Medicine, College of Medicine, Taipei Medical University, Taipei, Taiwan; f Dizziness and Balance Disorder Center, Taipei Medical University Hospital, Taipei Medical University, Taipei, Taiwan; g Department of Neurology, Shuang Ho Hospital, Taipei Medical University, Taipei, Taiwan; h Program in Specialty Crops and Metabolomics, Academy of Circular Economy, National Chung Hsing University, Nantou, Taiwan; i Agricultural Biotechnology Research Center, Academia Sinica, Taipei, Taiwan

**Keywords:** Cedrol, ultraviolet B (UVB) radiation, HaCaT, mitochondrial biogenesis, oxidative stress, tight junction proteins, metabolomics

## Abstract

**Context:**

Ultraviolet B (UVB) radiation is a key environmental contributor to skin photoaging, primarily by inducing oxidative stress, mitochondrial dysfunction, metabolic imbalance, and downregulation of tight junction (TJ) proteins. Cedrol, the major component of the essential oil from *Cunninghamia lanceolata* var. *konishii*, a tree species endemic to Taiwan, exhibits antioxidant properties. However, its restorative effects against UVB-induced skin damage have not been fully elucidated.

**Objective:**

In this study, HaCaT keratinocytes were used to evaluate the post-treatment effects of cedrol on UVB-induced damage to skin cells.

**Materials and methods:**

HaCaT cells were exposed to UVB irradiation followed by cedrol treatment. Cell viability, intracellular reactive oxygen species (ROS), mitochondrial membrane potential, ATP levels, mitochondrial biogenesis-related proteins (SIRT1, PGC-1α, Nrf2, TFAM), and TJ proteins (ZO-1, occludin, claudin-3) were assessed. Additionally, ^1^H-NMR-based metabolomics was conducted to evaluate UVB-induced metabolic changes.

**Results:**

Cedrol significantly improved cell viability post-UVB exposure, decreased intracellular reactive oxygen species (ROS), and restored mitochondrial membrane potential and ATP levels. It also upregulated mitochondrial biogenesis-related proteins (SIRT1, PGC-1α, Nrf2, and TFAM) and maintained TJ protein expression (ZO-1, occludin, and claudin-3), thereby preserving epithelial barrier integrity. Furthermore, ^1^H-NMR-based metabolomics revealed that cedrol mitigated UVB-induced metabolic disturbances, particularly in amino acid and energy pathways.

**Discussion and conclusion:**

Cedrol alleviates UVB-induced cellular damage by modulating mitochondrial function and metabolic homeostasis, indicating its potential as a natural agent for promoting skin recovery after UV exposure.

## Introduction

The skin is the largest organ of the human body and serves as the first line of defense against external environmental stimuli. However, with increasing age, the functional characteristics and normal structure of the skin gradually decline. Intrinsic aging is primarily genetically determined and represents the natural physiological changes that occur over time, whereas extrinsic aging is related to the cumulative effects of environmental factors such as solar radiation, smoking, pollution, nutrition, and lifestyle (Yaar et al. 2002). Among these, environmental insults such as ultraviolet (UV) radiation can damage the skin and accelerate skin aging by promoting loss of elasticity, a process known as photoaging (Oh et al. 2023). Ultraviolet radiation is classified by wavelength into UVA (320–400 nm), UVB (280–320 nm), and UVC (200–280 nm). Notably, UVB can penetrate the epidermis, causing both acute and chronic photodamage, inducing oxidative stress and DNA damage in cells, thereby promoting skin aging and even carcinogenesis (Kulms et al. 2002; Yang et al. 2023).

Skin barrier function depends on a multilayered architecture comprising the stratum corneum and intercellular tight junctions (TJs). TJs are primarily localized in the granular layer of the epidermis, where they play essential roles in regulating transepidermal water loss and preventing the infiltration of external substances (Svoboda et al. 2016). Key TJ proteins, including occludin, claudin-1, and zonula occludens-1 (ZO-1), are critical for maintaining intercellular cohesion and barrier integrity (Brandner et al. 2002). Skin aging, driven by both intrinsic and extrinsic factors such as UV radiation, is associated with physiological changes that compromise barrier function (Jin et al. 2016). In particular, UVB exposure has been shown to disrupt TJ structure, resulting in reduced barrier integrity and heightened skin sensitivity (Yuki et al. 2011). Accordingly, natural compounds that preserve or restore TJ integrity represent promising candidates for skin barrier protection and anti-photoaging strategies.

Mitochondria, often referred to as the powerhouses of the cell, are central to ATP production *via* the tricarboxylic acid (TCA) cycle and the electron transport chain (ETC) (Luo et al. 2022; San-Millan 2023). They are also a major source of reactive oxygen species (ROS), and mitochondrial dysfunction is closely linked to oxidative stress and metabolic imbalance. As such, mitochondria are considered key regulators of cellular metabolism and homeostasis (Chen et al. 2023), especially under stress conditions such as UVB irradiation. UVB exposure impairs mitochondrial function in skin cells by decreasing mitochondrial membrane potential, reducing ATP synthesis, and increasing ROS accumulation, ultimately triggering apoptosis and cellular damage. These deleterious effects are associated with impaired mitochondrial dynamics, including disruptions in biogenesis, fission, and fusion processes (He et al. 2022). Recent evidence suggests that preserving mitochondrial structure and function can mitigate UVB-induced damage, improve cell viability, and support skin barrier repair (Sreedhar et al. 2020; He et al. 2022). Mitochondrial biogenesis (MB), the process through which cells increase both the number and functional capacity of mitochondria, necessitates the coordinated replication of mitochondrial DNA (mtDNA), transcription, and synthesis of mitochondrially encoded proteins (Alattar et al. 2022). The transcriptional coactivator PGC-1α is a central regulator of MB and is modulated by pathways involving AMP-activated protein kinase (AMPK) and silent information regulator 1 (SIRT1). Therefore, activation of PGC-1α, SIRT1, and related signaling cascades may serve as a promising strategy for enhancing mitochondrial function and delaying skin photoaging. Overall, mitochondria represent critical targets for mitigating UVB-induced photodamage and restoring epidermal homeostasis.

*Cunninghamia lanceolata* var. *konishii*, a tree species endemic to Taiwan, yields essential oil from its heartwood that is rich in bioactive compounds, including cedrol, *α*-pinene, *α*-cedrene, and *α*-terpineol (Cheng et al. 2011). Among these, cedrol is the predominant constituent, accounting for up to 78% of the essential oil content (Hsiao et al. 2024). Cedrol has been reported to exhibit a range of biological activities, including antioxidant, anti-inflammatory, and anticancer properties (Chang et al. 2020; Sakhaee et al. 2020), and recent findings suggest its potential to restore intestinal barrier function (Xu et al. 2025). These properties, particularly their antioxidant, anti-inflammatory, and barrier-protective activities, are key mechanisms for alleviating photoaging and cellular stress-induced damage. Although direct studies on the effects of cedrol against UV-induced skin damage remain limited, the existing evidence provides a strong rationale for investigating its restorative potential in this context.

In this study, we aim to evaluate the potential of cedrol to reverse or mitigate existing UV-induced damage, using UVB irradiation as the primary cellular stress model. UVB, being more energetic than UVA, reliably induces acute and quantifiable epidermal damage in HaCaT keratinocytes, including DNA damage, cellular apoptosis, oxidative stress, mitochondrial dysfunction, and impaired skin barrier function. This makes UVB an efficient *in vitro* tool for assessing a drug’s restorative potential (Colombo et al. 2017). In contrast, UVA-induced damage primarily affects deeper dermal collagen and causes chronic oxidative stress, often requiring more complex co-culture systems or more prolonged treatment periods (Wang et al. 2019), which are less suitable for studies focusing on keratinocyte mitochondrial and barrier function. Therefore, in this study, we investigated the restorative effects of cedrol on UVB-induced damage in HaCaT cells, with particular attention to its modulation of mitochondrial function and tight junction protein expression, which are critical for reestablishing skin barrier integrity after UVB exposure.

## Materials and methods

### Reagents and materials

High-glucose DMEM (Gibco, USA; 12100-046), fetal bovine serum (Gibco, USA), 100 mM sodium pyruvate (Corning, USA; 25-000-CI), penicillin–streptomycin (Gibco, USA; 15140-122), ATP detection assay kit–luminescence (Cayman Chemical, USA), DMSO (Sigma-Aldrich, Germany; D2650), methanol-d_4_ (Sigma-Aldrich, Germany; 151947-10G-GL), deuterium oxide (Sigma-Aldrich, Germany; 151882-100G), 3-(trimethylsilyl)propionic-2,2,3,3-d_4_ acid sodium salt (TSP) (Sigma-Aldrich, Germany; 269913-1G), Enhanced Cell Counting Kit-8 (Elabscience, China; E-CK-A362), Pierce™ RIPA buffer (Thermo Fisher Scientific, USA), protease inhibitor cocktail (GoalBio, Taiwan; HC100-007), phosphatase inhibitor cocktail (GoalBio, Taiwan; HC100-008), bovine serum albumin (Gibco, USA), Bio-Rad protein assay dye reagent concentrate (Bio-Rad, USA), 5× protein sample dye (GeneMark, Taiwan; GM47-b), mitochondrial membrane potential assay kit (JC-1) (Abbkine, China; KTA4001), L-amino acids (Sigma-Aldrich, USA; LLA21), resveratrol (≥99% HPLC, Sigma-Aldrich, Germany; R5010), H_2_DCFDA (2′,7′-dichlorofluorescin diacetate) (Sigma-Aldrich, Germany; D6883).

### Essential oil extraction and cedrol purification

The *Cunninghamia lanceolata* var. *konishii* wood used in this study was generously provided by Dr. Min Jay Chung of the Experimental Forest Management Office, National Taiwan University, Nantou County, Taiwan. The collected wood was air-dried and cut into small chips (approximately 1–2 cm in size) before extraction. Essential oil was extracted from *Cunninghamia lanceolata* var. *konishii* wood using hydrodistillation. Briefly, 200 g of dried wood was placed in a round-bottom flask containing 1 L of distilled water. The mixture was subjected to steam distillation for 6 h, after which the essential oil (CKEO) was collected. High-purity cedrol was subsequently isolated by recrystallization. The purity of the obtained cedrol was determined to be 96.4%, as confirmed by gas chromatography-mass spectrometry (GC-MS) and proton nuclear magnetic resonance spectroscopy (^1^H-NMR) (Xu et al. 2025). The complete chemical composition of the essential oil is provided in Supplementary Table 1.

### Cell culture

HaCaT cells, an immortalized human keratinocyte cell line, were kindly provided by Dr. P. S. Lai (National Chung Hsing University, Taichung, Taiwan). The HaCaT cell line (CVCL_0038) was obtained from AddexBio (Catalog #: T0020001, San Diego, CA, USA). Cells were cultured in high-glucose Dulbecco’s Modified Eagle Medium (DMEM; Gibco, USA; 12100-046) supplemented with 10% (v/v) fetal bovine serum (FBS), 1% (v/v) penicillin–streptomycin (Gibco, USA; 15140-122), and 3.7 g/L sodium bicarbonate (NaHCO_3_). The cells were maintained in a humidified incubator at 37 °C with 5% CO_2_.

### UVB irradiation and drug treatment

HaCaT cells were seeded in appropriate plates at densities optimized for each assay and incubated for 24 h. After incubation, cells were washed with 1× phosphate-buffered saline (PBS) and exposed to UVB irradiation at a dose of 100–200 mJ/cm^2^ using a UV crosslinker (CL-3000, Analytik Jena, Germany) in PBS. Immediately after UVB exposure, PBS was replaced with fresh culture medium containing the test compounds (CKEO, cedrol, or resveratrol as a positive control) dissolved in medium with 0.1% DMSO. Cells were further incubated for 24 h before downstream assays.

### Cell viability assay

Cell viability was assessed using the Cell Counting Kit-8 (CCK-8; WST-8), which contains the water-soluble tetrazolium salt WST-8 [2-(2-methoxy-4-nitrophenyl)-3-(4-nitrophenyl)-5-(2,4-disulfophenyl)-2H-tetrazolium, monosodium salt]. In viable cells, WST-8 is reduced by cellular dehydrogenases to produce an orange-colored formazan dye. The intensity of the color, measured by absorbance at 450 nm, is directly proportional to the number of living cells.

For cell viability assays, the culture medium was replaced with fresh medium containing 1% (v/v) Enhanced Cell Counting Kit-8 reagent. Cells were then incubated at 37 °C for 1 h, and absorbance at 450 nm was measured using a microplate spectrophotometer (μQuant, BioTek, USA) to determine cell viability.

$$\text{cell viability }\left(\%\right)\;=\;\left({\text{Treatment OD}}_{450}/{\text{Control OD}}_{450}\right)\;\times 100$$

### Intracellular reactive oxygen species (ROS) assay

Intracellular reactive oxygen species (ROS) accumulation in HaCaT cells was assessed using the fluorescent probe 2′,7′-dichlorofluorescin diacetate (H_2_DCF-DA), with minor modifications based on previously reported methods (Masaki et al. 2009). For intracellular ROS assays, the medium was removed, and the cells were washed twice with 1× PBS. H_2_DCF-DA was dissolved in PBS to a final concentration of 30 μM and added to each well. After incubation for 30 min at 37 °C in the dark, fluorescence intensity was measured using a multilabel microplate reader (Hidex, Finland) at excitation/emission wavelengths of 485/535 nm. The percentage of ROS generation was calculated using the following formula:

$$\text{ROS production }\left(\%\right)\;=\;({\text{Treatment A}}_{485}{/}_{535}/{\text{Control A}}_{485}{/}_{535})\;\times \text{ 1}00$$

### ATP assay

The intracellular ATP content of HaCaT cells was determined using a luminescence-based ATP assay, following the method described by Li et al. (2023). ATP levels were measured using an ATP detection assay kit–luminescence (Cayman Chemical, USA) according to the manufacturer’s instructions. Luminescence was detected at 535 nm using a multilabel microplate reader (Hidex, Finland). ATP concentrations were calculated based on standard curves and normalized to the control group, which was set as 100%.

### Mitochondrial membrane potential assay

Mitochondrial function in differentiated HaCaT cells was assessed by measuring the mitochondrial membrane potential (Δψm) using JC-1 dye [5,5′,6,6′-tetrachloro-1,1′,3,3′-tetraethylbenzimidazolylcarbocyanine iodide], a cationic fluorescent probe that accumulates in mitochondria in a membrane potential–dependent manner. The experimental procedure was adapted from a previously described method (Sharma et al. 2020). Mitochondrial membrane potential was evaluated using the JC-1 Mitochondrial Membrane Potential Assay Kit (Abbkine, China; KTA4001) following the manufacturer’s protocol. Fluorescence imaging was performed with an upright fluorescence microscope (ECLIPSE Ci, Nikon, Japan) equipped with a 40× objective lens. Images were captured using a microscopic imaging system (SGHD-3.6, SAGE Vision, Taiwan) connected to SG Image V2.3 software (Taiwan). Quantification of fluorescence intensity and channel merging was conducted using ImageJ software (National Institutes of Health, USA).

### Western blot analysis

Western blotting was conducted to evaluate the expression of mitochondrial biogenesis-related and tight junction proteins in HaCaT cells, with modifications based on previously described protocols (He et al. 2022; Wang et al. 2024).

For protein extraction, cells were washed with cold PBS, scraped, and lysed in RIPA buffer (Pierce™, Thermo Fisher Scientific, USA) supplemented with 1% protease and phosphatase inhibitor cocktails. Lysates were incubated on ice with intermittent vortexing, then centrifuged at high speed. Supernatants were collected and stored at −80 °C. Protein concentrations were determined using the BCA assay and adjusted to 90 μg/mL. Samples were denatured at 37 °C for 5 min prior to electrophoresis.

Proteins were separated on 12% SDS-PAGE gels and transferred to PVDF membranes (Revvity, USA) at 300 mA for 1–2 h. Membranes were washed with TBST and blocked with EZBlocker (GenePure, Taiwan) for 10 min at room temperature. Membranes were then incubated overnight at 4 °C with primary antibodies targeting: Occludin (1:10,000; 27260-1-AP, Proteintech, China), TFAM (1:20,000; 22586-1-AP, Proteintech, China), PGC-1α (1:10,000; 66369-1-lg, Proteintech, China), Claudin-1 (1:500; 51-9000, Invitrogen, USA), Claudin-3 (1:1,000; 34-1700, Invitrogen, USA), AMPKα1 (1:1,000; 07-350, Millipore Sigma, USA), *p*-AMPKα (1:1,000; 2535, Cell Signaling Technology, USA), SIRT1 (1:1,000; 9475s, Cell Signaling Technology, USA), Nrf2 (1:1,000; 12721, Cell Signaling Technology, USA), β-actin (1:5,000; SC-47778, Santa Cruz, UK)

Following primary antibody incubation, membranes were washed with TBST and incubated with species-specific HRP-conjugated secondary antibodies: goat anti-mouse IgG (1:10,000; ARG65350, Arigo Biolaboratories, China) or rabbit IgG (1:10,000; abs-22-200, Asia Bioscience, Taiwan) for 2 h at room temperature with gentle shaking. After final washes with TBST, immunoreactive bands were visualized using WesternBright™ ECL substrate (Advansta, USA) and detected *via* chemiluminescence.

### Metabolic profiling by ^1^H NMR spectroscopy

Metabolic profiling of HaCaT cell monolayers was performed using a nuclear magnetic resonance (NMR) spectrometer (Avance III-400, Bruker, USA). The ^1^H NMR protocol was adapted from Xu et al. (Xu et al. 2025).

For sample preparation, cells were washed with ice-cold PBS, scraped, and collected into centrifuge tubes. After centrifugation to remove residual PBS, the resulting pellets were either stored at −80 °C or used immediately for metabolite extraction. For extraction, pellets were treated with ice-cold 70% methanol, followed by ultrasonication and vortexing on ice. The mixture was centrifuged, and the supernatant was collected and dried under vacuum. The dried metabolites were reconstituted in a 1:1 mixture of deuterium oxide (D_2_O) and methanol-d_4_ (MeOD) containing 0.0002% 3-(trimethylsilyl)propionic-2,2,3,3-d_4_ acid sodium salt (TSP) as an internal standard. A volume of 600 μL of each sample was transferred into 5 mm NMR tubes for spectral acquisition.

All spectra were acquired using the Avance III-400 NMR spectrometer operating at a proton frequency of 400 MHz and a temperature of 300 K. MeOD was used as the internal lock solvent. Acquisition parameters for ^1^H spectra included a 90° pulse angle, 1000 scans, a spectral width of 20 ppm, and a relaxation delay of 6.5 s. Spectra were processed using TopSpin software (version 3.5pl7, Bruker, Germany). TSP was used for both chemical shift calibration (0.00 ppm) and normalization of metabolite signal integrals.

Integrated spectral data were exported to Excel, converted to CSV format, and imported into MetaboAnalyst 6.0 (https://www.metaboanalyst.ca/) for statistical analysis. Data were normalized using autoscaling. Initial exploration of metabolic variation across groups was conducted using unsupervised principal component analysis (PCA). To refine group separation and minimize systematic and environmental noise, supervised sparse partial least squares discriminant analysis (sPLS-DA) was performed. Comparisons among the four experimental groups (control, cedrol, UVB, and UVB + cedrol) were used to identify metabolites that were significantly altered, based on loading plots of the major predictive components. Hierarchical clustering and heatmap analyses were performed to visualize patterns of metabolite abundance across samples. Heatmaps were generated using Euclidean distance as the distance measure and Ward’s linkage method for clustering. All analyses, including PCA, sPLS-DA, loading plots, and heatmaps, were conducted using default parameters in MetaboAnalyst 6.0 unless otherwise specified. Metabolite identification was conducted using chemical shift data referenced against the Human Metabolome Database (HMDB, http://www.hmdb.ca), the Biological Magnetic Resonance Data Bank (BMRB, http://www.bmrb.wisc.edu), and relevant literature (Carneiro et al. 2019).

### Statistical analysis

All data are presented as mean ± standard deviation (SD) from at least three independent experiments (*n* ≥ 3), unless otherwise specified. The exact *n* values for each experiment are indicated in the corresponding figure legends. Statistical analyses were performed using GraphPad Prism 8.0 software (Dotmatics, USA). Comparisons between two groups were conducted using unpaired *t*-tests, while comparisons among multiple groups were evaluated using one-way analysis of variance (ANOVA) followed by Tukey’s honestly significant difference (HSD) post hoc test. A *p*-value < 0.05 was considered statistically significant.

## Result

### Cell viability and ROS generation in keratinocytes induced by UVB irradiation

**Figure 1.**
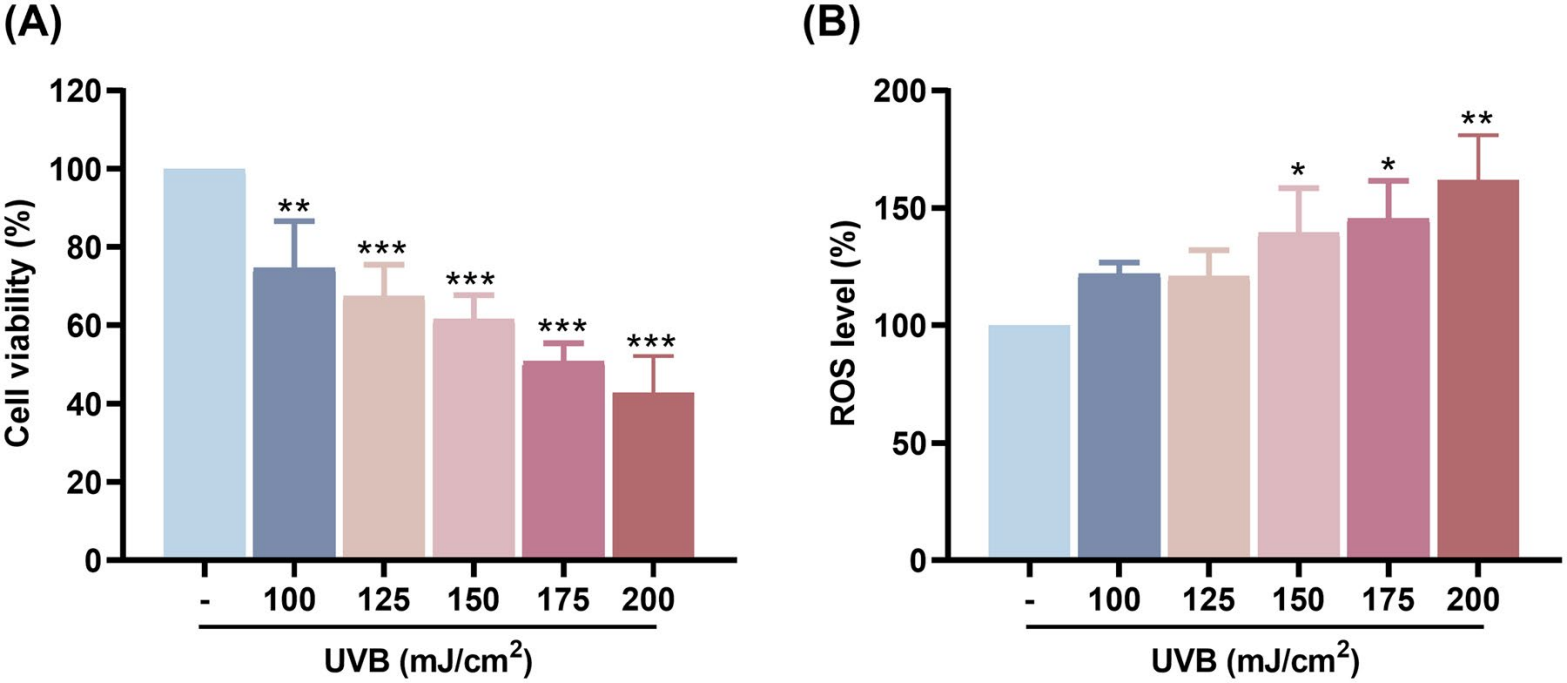
Effects of UVB irradiation on HacaT cell viability and intracellular reactive oxygen species (ROS) levels *in vitro*. (A) The cell viability of HaCaT cells was exposed to 100-200 mJ/cm² UVB irradiation. (B) Intracellular ROS levels were measured using a DCFH-DA fluorescent probe following UVB exposure. Data are presented as mean ± SD (*n* = 3). Statistical analysis was performed using one-way ANOVA followed by Tukey’s HSD post hoc test. * *p* < 0.05, ** *p* < 0.01, *** *p* < 0.001, **** *p* < 0.0001, compared with control group.

To assess the cytotoxic effects of UVB irradiation on HaCaT cells, a CCK-8 assay was conducted. The results show that UVB exposure at doses ranging from 100 to 200 mJ/cm^2^ resulted in a dose-dependent decrease in cell viability. A significant reduction was observed at doses ≥125 mJ/cm^2^ (***p < 0.001), indicating that high-intensity UVB exerts cytotoxic effects on HaCaT cells (Figure 1(A)). Notably, irradiation at 150 mJ/cm^2^ led to an approximate 60% reduction in cell viability. To evaluate UVB-induced oxidative stress, intracellular reactive oxygen species (ROS) levels were measured. As illustrated in Figure 1(B), UVB exposure significantly increased ROS production at 150, 175, and 200 mJ/cm^2^ (*p < 0.05, **p < 0.01), further confirming a dose-dependent oxidative response. Taken together, these results demonstrate that UVB irradiation at 150 mJ/cm^2^ markedly reduces cell viability and elevates intracellular ROS levels, making it an appropriate dose for establishing a UVB-induced photoaging model in keratinocytes.

### Effects of Cunninghamia lanceolata essential oil and its major component cedrol on alleviating UVB-induced cytotoxicity in keratinocytes

**Figure 2.**
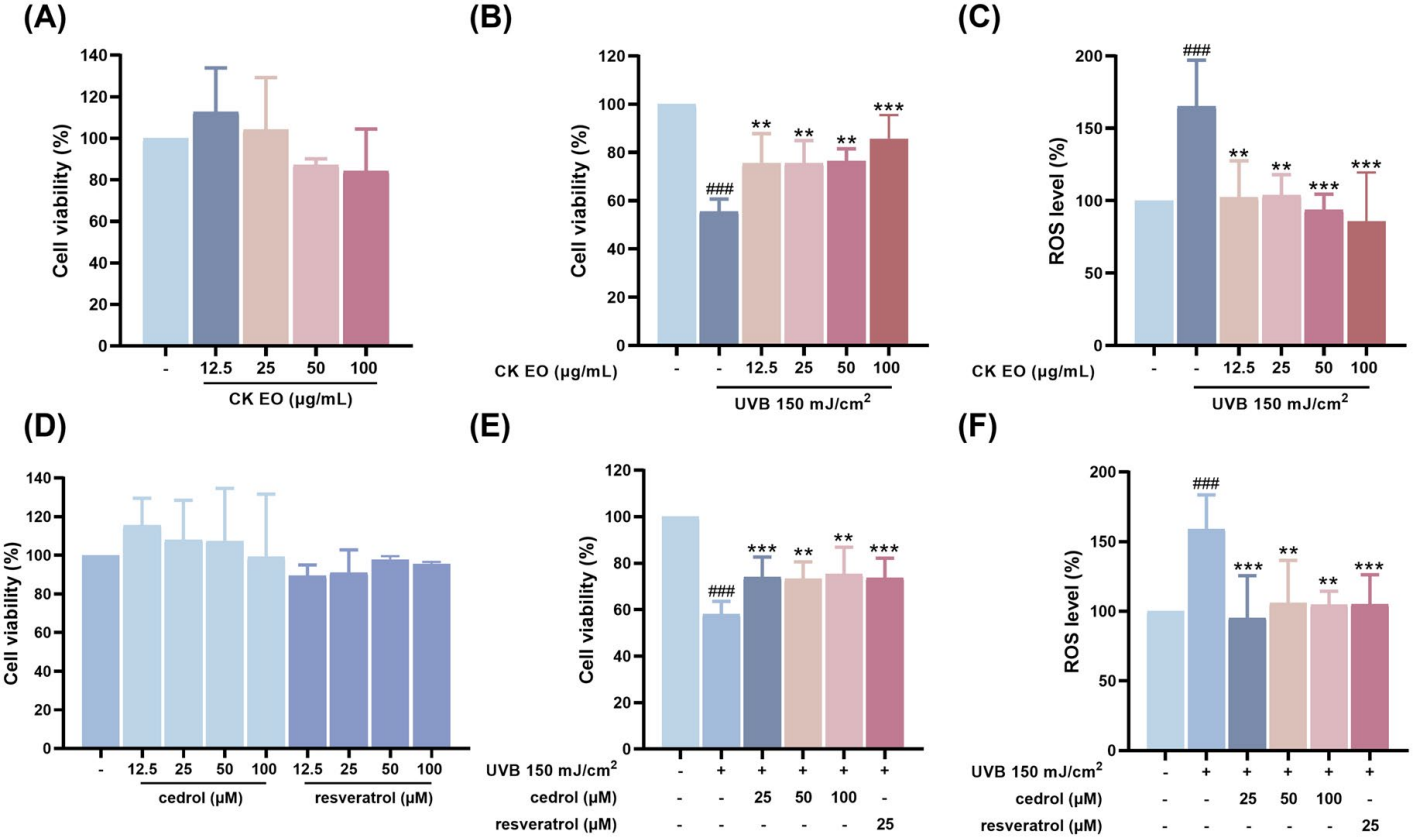
Effects of CK essential oil (CK EO), cedrol, and resveratrol on cell viability and UVB-induced oxidative stress in HaCaT cells. (A) Cell viability after treatment with various concentrations of CK EO. (B) Restorative effects of CK EO against UVB-induced reduction in cell viability. (C) Effects of CK EO on UVB-induced intracellular ROS production. (D) Cell viability following treatment with various concentrations of cedrol or resveratrol. (E) Restorative effects of cedrol against UVB-induced cell damage. (F) Effects of cedrol on UVB-induced ROS generation. Data are presented as mean ± SD (*n* = 3). Statistical analysis was performed using one-way ANOVA followed by Tukey’s HSD post hoc test. ###*p* < 0.001 vs. control group; **p < 0.01, ***p < 0.001, ****p < 0.0001 vs. UVB group. Resveratrol (25 μM) was used as a positive control.

To evaluate the restorative effects of *Cunninghamia lanceolata* essential oil (CKEO) and its major constituent, cedrol, on HaCaT cells following UVB exposure, cell viability was assessed using the CCK-8 assay. Resveratrol, a naturally occurring polyphenolic compound with well-documented antioxidant and anti-inflammatory properties, was included as a positive control due to its established efficacy in reducing UVB-induced ROS production and mitigating cellular damage (Vitale et al. 2013). The results show that CKEO (≤100 μg/mL), cedrol (≤100 μM), and resveratrol did not exert significant cytotoxic effects on HaCaT cells (Figure 2(A,D)). In contrast, treatment with CKEO (12.5–100 μg/mL) and cedrol (25–100 μM) following UVB exposure significantly improved cell viability compared to UVB-only treated cells (Figure 2(B,E)). Intracellular reactive oxygen species (ROS) levels were measured using the fluorescent probe H_2_DCF-DA. As shown in Figure 2(C,F), UVB irradiation significantly elevated ROS levels in HaCaT cells compared to the control group. However, treatment with CKEO and cedrol effectively reduced UVB-induced ROS accumulation, restoring ROS levels to near-control values. These findings suggest that CKEO and its major active component, cedrol, mitigate UVB-induced cytotoxicity and oxidative stress in HaCaT keratinocytes, supporting their potential as restorative agents for alleviating photoaging-related skin damage.

### Cedrol ameliorates UVB-induced mitochondrial damage in keratinocytes

**Figure 3.**
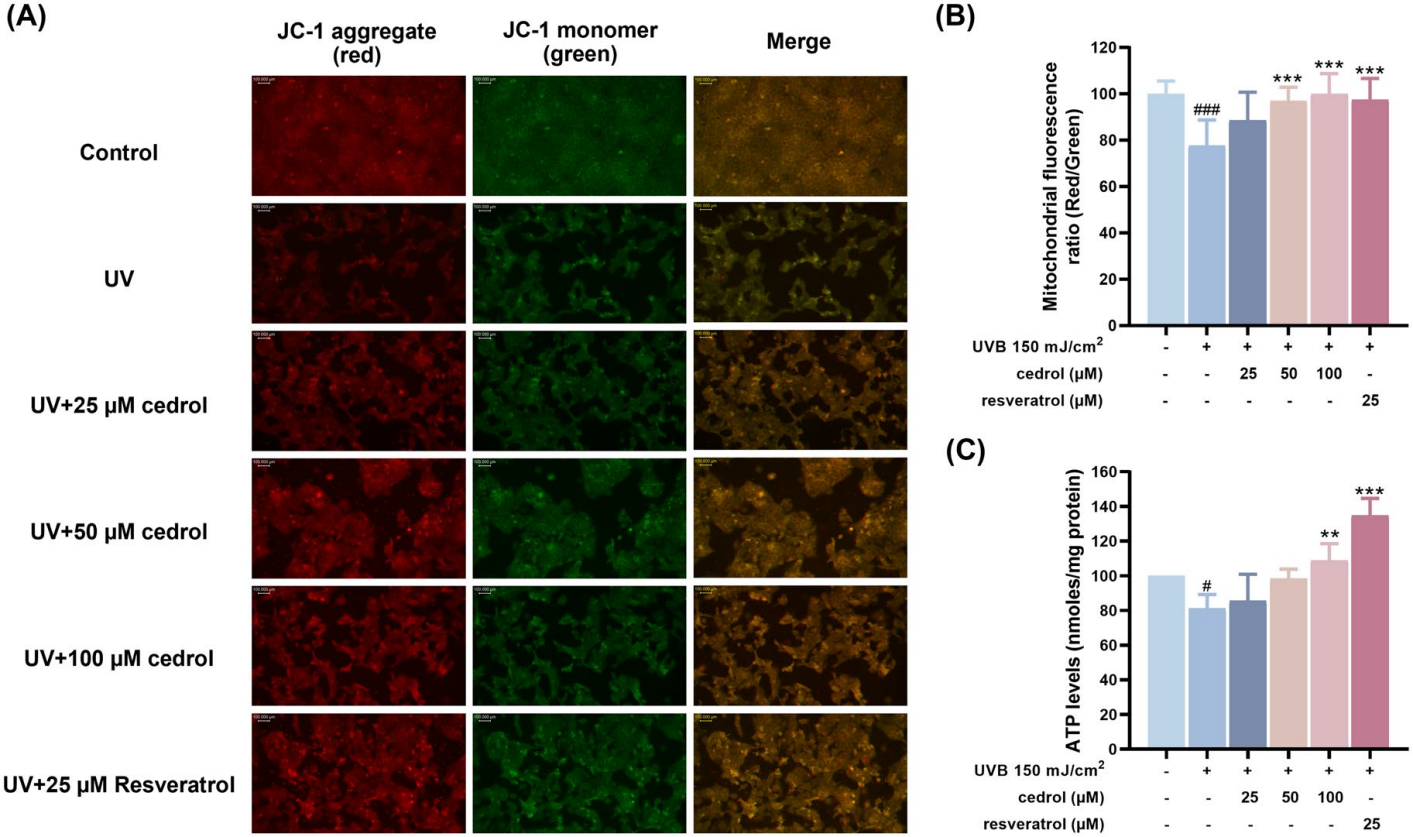
Effects of cedrol on mitochondrial membrane potential and ATP production in UVB-irradiated HaCaT cells. (A) Representative fluorescence images of mitochondrial membrane potential assessed by JC-1 staining. Red fluorescence indicates JC-1 aggregates (healthy mitochondria), and green fluorescence indicates JC-1 monomers (depolarized mitochondria). Nuclei were stained with DAPI. Images were captured using a fluorescence microscope with a 40× objective. (B) Quantification of mitochondrial membrane potential based on the red/green fluorescence ratio. (C) Intracellular ATP levels were measured following treatment with cedrol. Data are presented as mean ± SD (*n* = 3). Statistical analysis was performed using one-way ANOVA followed by Tukey’s HSD post hoc test. #*p* < 0.05, ###*p* < 0.001 vs. the control group; **p < 0.01, ***p < 0.001 vs. UVB group. Resveratrol (25 μM) was used as a positive control.

To investigate whether cedrol exerts restorative effects on mitochondria, changes in mitochondrial membrane potential (Δψm) in HaCaT cells following UVB irradiation were assessed using JC-1 staining. JC-1 is a cationic dye that accumulates in mitochondria in a membrane potential-dependent manner. In healthy, polarized mitochondria, JC-1 forms aggregates that emit red fluorescence (585/590 nm), whereas in depolarized mitochondria, JC-1 remains as monomers in the cytosol, emitting green fluorescence (510/527 nm). Thus, the red to green fluorescence (red/green) serves as an indicator of mitochondrial functional status. As shown in Figure 3(A), the control group exhibited predominantly red fluorescence, indicative of intact mitochondrial membrane potential. UVB exposure caused a pronounced shift from red to green fluorescence, corresponding to a significant loss of Δψm, as evidenced by a decreased JC-1 red/green ratio. These findings confirm that UVB irradiation induces mitochondrial dysfunction in keratinocytes. Treatment with cedrol (25–100 μM) dose-dependently restored mitochondrial membrane potential, as shown by increased red fluorescence and elevated JC-1 red/green ratios. These effects were comparable to those observed with the positive control, resveratrol (25 μM) (Figure 3(B)), suggesting that cedrol mitigates UVB-induced mitochondrial depolarization. To further evaluate mitochondrial function, intracellular ATP levels were measured. UVB exposure significantly reduced ATP production in HaCaT cells, whereas cedrol treatment restored ATP levels in a dose-dependent manner. Notably, treatment with 100 μM cedrol or resveratrol significantly increased intracellular ATP concentrations to levels comparable with the control group (Figure 3(C)). Collectively, these findings demonstrate that cedrol alleviates UVB-induced mitochondrial damage in keratinocytes by preserving membrane potential and restoring cellular energy metabolism. These results support the potential of cedrol as a restorative and anti-photoaging agent targeting mitochondrial function.

### Cedrol improves mitochondrial biogenesis in UVB-irradiated keratinocytes

**Figure 4.**
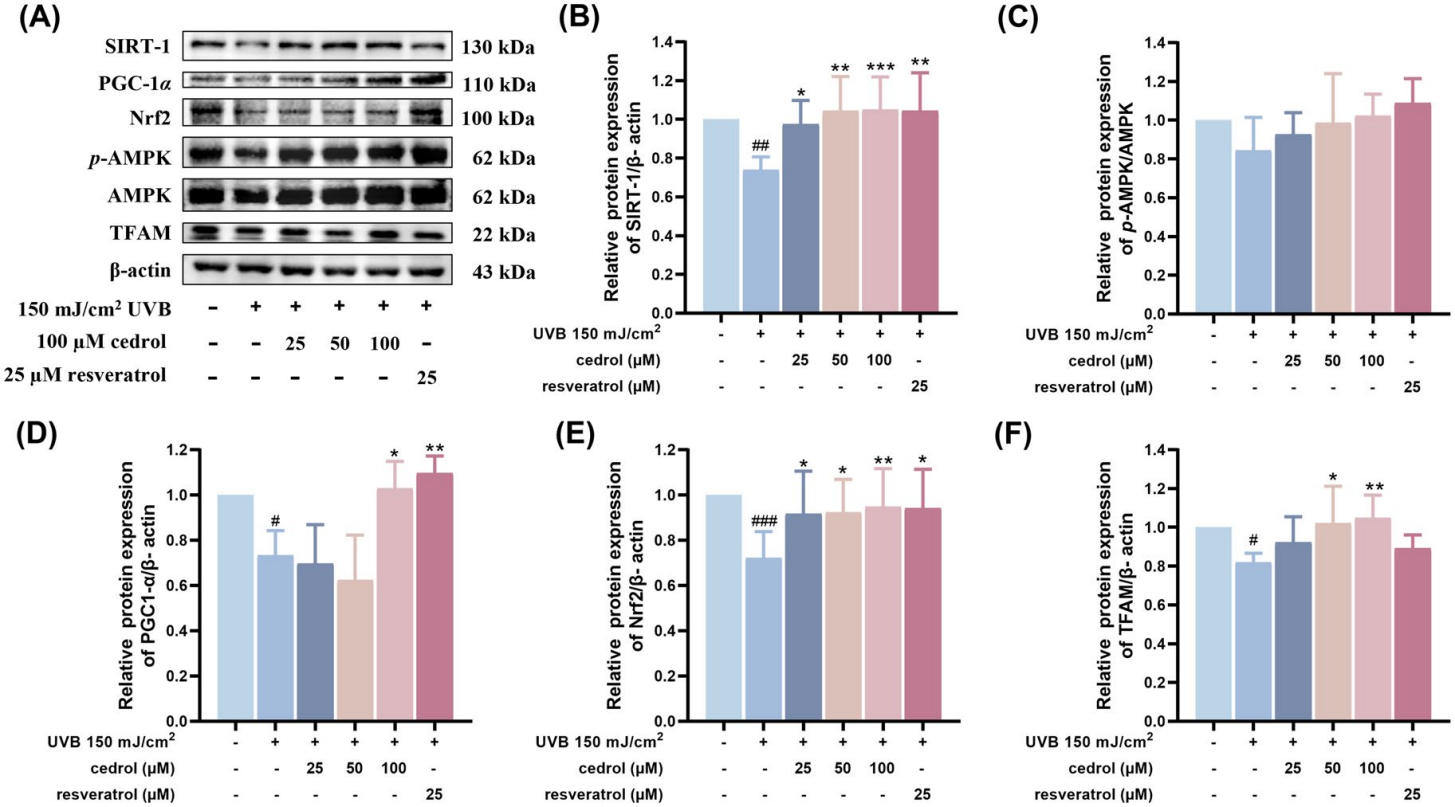
The effects of cedrol on the expression of mitochondrial biogenesis protein in UVB-irradiated HaCaT cells. (A) Western blotting visual image of SIRT-1, PGC-1α, Nrf2, p-AMPK, AMPK, and TFAM. Relative protein expression levels of (B) SIRT-1, (C) p-AMPK/AMPK, (D) PGC-1α, (E) Nrf2, and (F) TFAM in the HaCaT cells. β-actin was used as the protein loading control. Data are presented as mean ± SD (*n* = 3). Statistical analysis was performed using one-way ANOVA followed by Tukey’s HSD post hoc test. #*p* < 0.05, ##*p* < 0.01, ###*p* < 0.001 vs. the control group; *p < 0.05, **p < 0.01, ***p < 0.001 vs. UVB group. Resveratrol (25 μM) was used as a positive control.

To investigate whether cedrol alleviates UVB-induced cellular damage by regulating mitochondrial function-related signaling pathways, the expression levels of key regulatory proteins, including SIRT1, PGC-1α, Nrf2, *p*-AMPK/AMPK, and TFAM, were evaluated. UVB irradiation markedly reduced the expression of SIRT1, PGC-1α, Nrf2, and TFAM compared to the non-irradiated control group, while *p*-AMPK/AMPK levels remained unchanged (Figure 4(A–F)). Treatment with cedrol (25–100 μM) significantly restored the expression of SIRT1, PGC-1α, Nrf2, and TFAM in a dose-dependent manner, with prominent effects observed at 50 and 100 μM (Figure 4(B,D,E,F)). The *p*-AMPK/AMPK ratio showed no statistically significant differences among groups (Figure 4(C)), suggesting that cedrol does not exert its effects through AMPK pathway activation. Collectively, these findings indicate that cedrol counteracts UVB-induced suppression of proteins critical for mitochondrial biogenesis and antioxidant defense, primarily through modulation of the SIRT1/PGC-1α/Nrf2/TFAM signaling axis.

### Cedrol restores barrier function in UVB-irradiated keratinocytes

**Figure 5.**
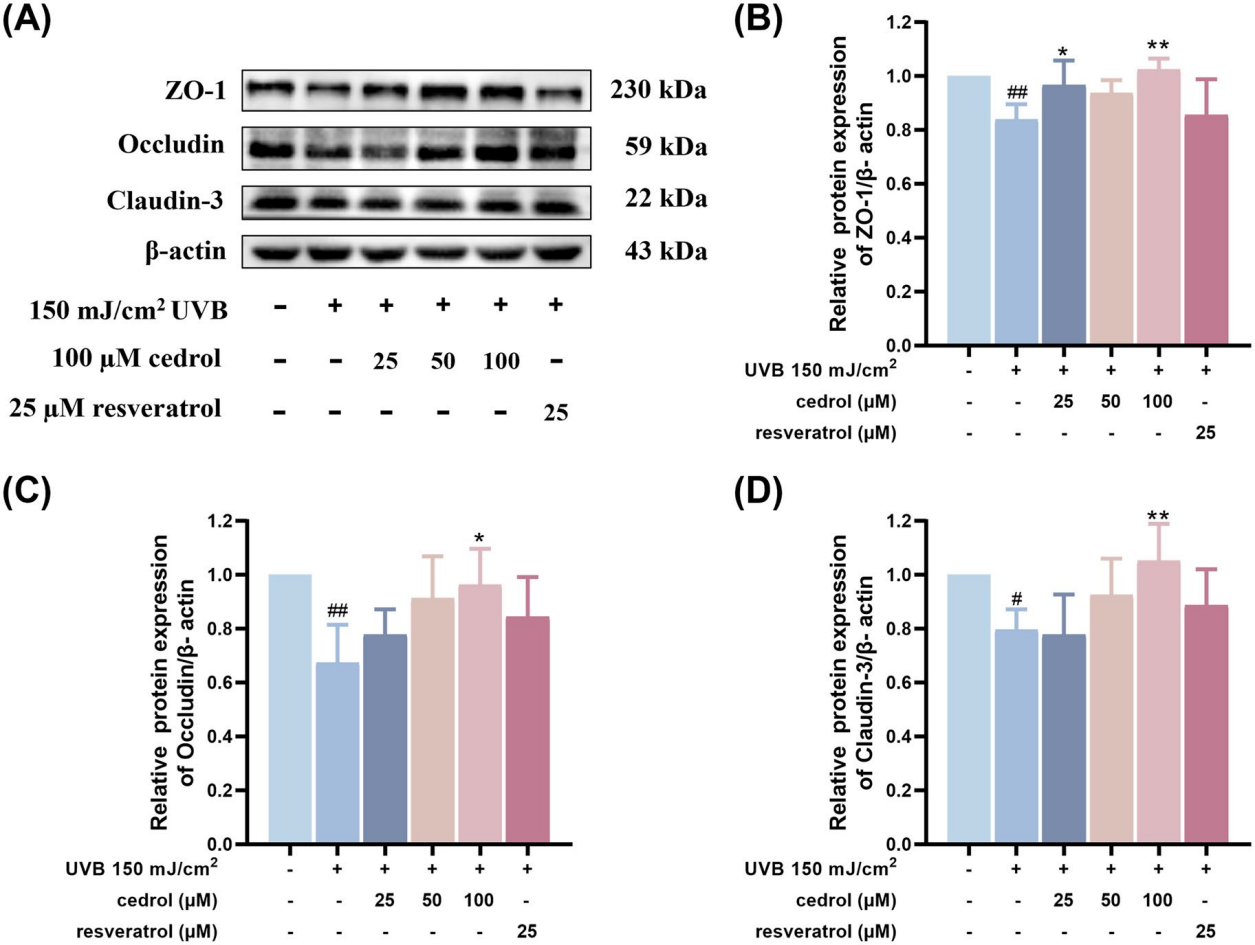
The effects of cedrol on the expression of tight junction protein in UVB-irradiated HaCaT cells. (A) Western blotting visual image of zo-1, occludin, and claudin-3. Relative protein expression of (B) ZO-1, (C) occludin, and (D) claudin-3 in different groups. β-actin was used as the protein loading control. Data are presented as mean ± SD (*n* = 4). Statistical analysis was performed using one-way ANOVA followed by Tukey’s HSD post hoc test. #*p* < 0.05, ##*p* < 0.01 vs. the control group; *p < 0.05, **p < 0.01 vs. UVB group. Resveratrol (25 μM) was used as a positive control.

To investigate whether cedrol can alleviate UVB-induced skin barrier damage, this study further examined the expression of tight junction proteins, including ZO-1, occludin, and claudin-3. As shown in Figure 5(A), UVB irradiation significantly decreased the protein expression of ZO-1, occludin, and claudin-3. However, treatment with cedrol restored the expression of these tight junction proteins in a dose-dependent manner (Figure 5(B–D)). In conclusion, these results suggest that cedrol effectively maintains the expression of tight junction proteins under UVB irradiation, thereby contributing to the preservation of skin barrier integrity. Cedrol may thus serve as a potential candidate for the prevention or repair of UVB-induced skin damage.

### ^1^H NMR analysis of metabolites in UVB-irradiated HaCaT cells

**Figure 6.**
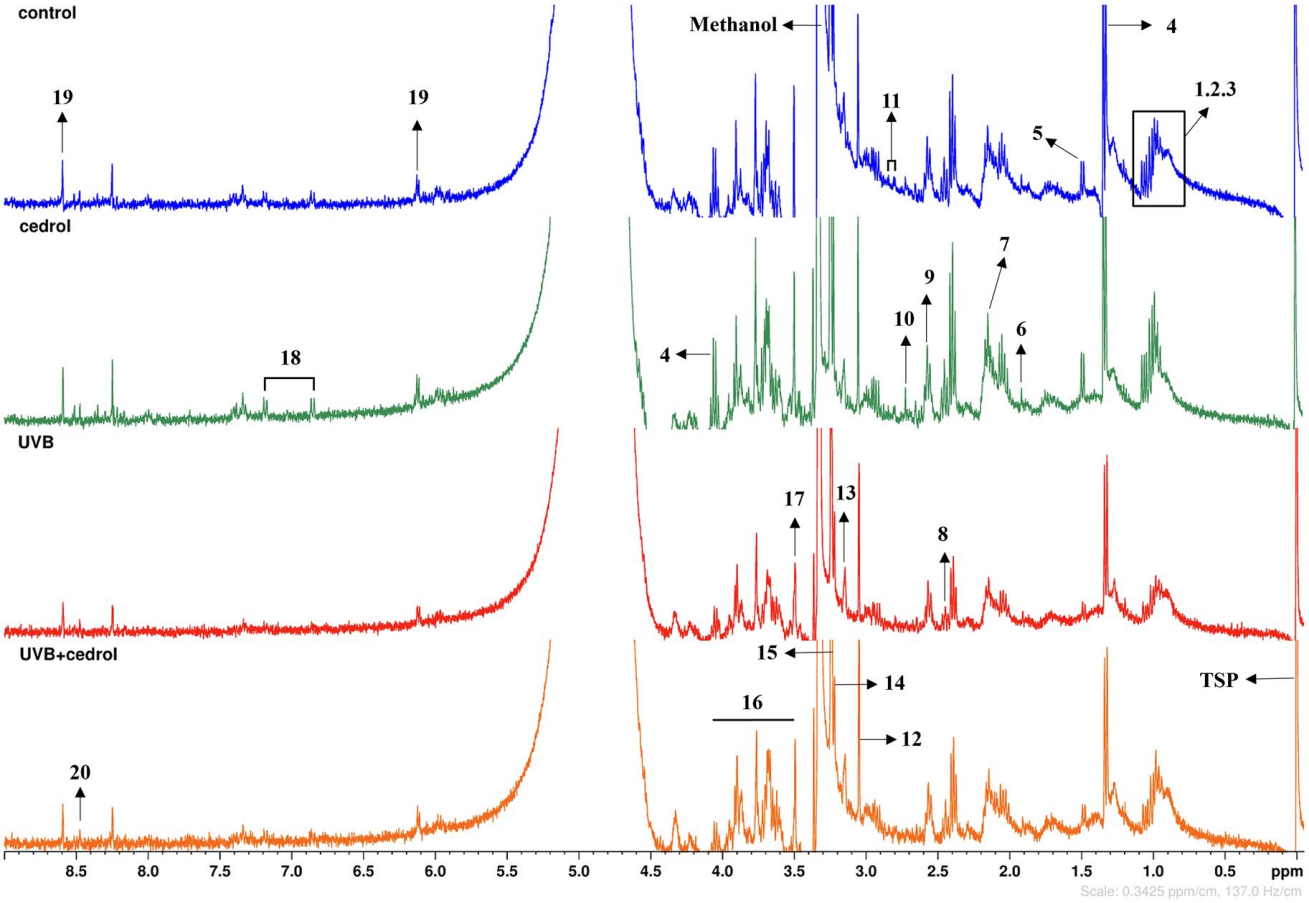
The identified metabolites of HacaT cells in 400 MHz ^1^H-NMR spectra. The characteristic peaks from the identified metabolites in the HaCaT cell are annotated with a corresponding number. All cell extractions are prepared in 650 μL D2O + MeOD (including 0.0002% TSP). Metabolites:1, leucine; 2, isoleucine; 3, valine; 4, lactate; 5, alanine; 6, acetate; 7, glutamine; 8, succinate; 9, glutamate; 10, sarcosine; 11, aspartate; 12, creatine; 13, choline; 14, *o*-phosphocholine; 15, *sn*-glycerophosphocholine; 16, glucose; 17, glycine; 18, tyrosine; 19, ATP; 20, formate.

To evaluate whether cedrol can ameliorate UVB-induced metabolic disturbances, a ^1^H-NMR-based metabolomic analysis was performed. Figure 6 presents the ^1^H-NMR spectra of HaCaT cells under different treatment conditions. A total of 20 intracellular metabolites were identified based on chemical shift data reported in previous literature and reference databases, including the Human Metabolome Database (HMDB, http://www.hmdb.ca) and the Biological Magnetic Resonance Data Bank (BMRB, http://www.bmrb.wisc.edu), as summarized in Table 1 (Carneiro et al. 2019). Compared with the control group, UVB irradiation induced pronounced alterations in several metabolite peaks, indicating significant changes in the cellular metabolic profile. Notably, the spectra of the UVB + cedrol treatment group more closely resembled those of the control group, suggesting a partial reversal of UVB-induced metabolic dysregulation. Furthermore, the cedrol-alone group exhibited spectra similar to those of the control group, implying that cedrol has minimal influence on basal cellular metabolism.

**Table 1.** The chemical shifts of different metabolites in HacaT cells were identified by ^1^H NMR.

NO.	Metabolite name	Chemical shift (ppm)	References
1	Leucine	0.98(t)	(Kostidis et al. 2017; Carneiro et al. 2019)
2	Isoleucine	0.95(t), 1.02(d), 3.6(d)	(Kostidis et al. 2017; Carneiro et al. 2019)
3	Valine	1.0(d), 1.06(d)	(Kostidis et al. 2017; Carneiro et al. 2019)
4	Lactate	1.33(d), 4.05(t)	(Kostidis et al. 2017; Carneiro et al. 2019)
5	Alanine	1.48(d)	(Kostidis et al. 2017; Carneiro et al. 2019)
6	Acetate	1.90(s)	(Kostidis et al. 2017; Carneiro et al. 2019)
7	Glutamine	2.13(m), 2.45(m), 3.71(t)	(Kostidis et al. 2017; Carneiro et al. 2019)
8	Succinate	2.42(s)	(Kostidis et al. 2017; Carneiro et al. 2019)
9	Glutamate	2.04(m), 2.11(m), 2.54(m), 3.72(dd)	(Kostidis et al. 2017; Carneiro et al. 2019)
10	Sarcosine	2.71(s), 3.6(s)	(Kostidis et al. 2017; Carneiro et al. 2019)
11	Aspartate	2.84(m), 2.99(m), 3.97(m)	(Kostidis et al. 2017)
12	Creatine	3.04(s)	(Kostidis et al. 2017; Carneiro et al. 2019)
13	Choline	3.21(s)	(Kostidis et al. 2017; Carneiro et al. 2019)
14	*o*-Phosphocholine	3.23(s)	(Carneiro et al. 2019)
15	*sn*-Glycerophsphocholine	3.24(s)	(Kostidis et al. 2017)
16	Glucose	3.31-3.84(m), 4.58(d), 5.18(d)	(Kostidis et al. 2017; Carneiro et al. 2019)
17	Glycine	3.50(s)	(Kostidis et al. 2017; Carneiro et al. 2019)
18	Tyrosine	6.85(d), 7.18(d)	(Kostidis et al. 2017; Carneiro et al. 2019)
19	ATP	8.24(s), 8.51(s)	(Kostidis et al. 2017; Carneiro et al. 2019)
20	Formate	8.44(s)	(Kostidis et al. 2017; Carneiro et al. 2019)

**Figure 7.**
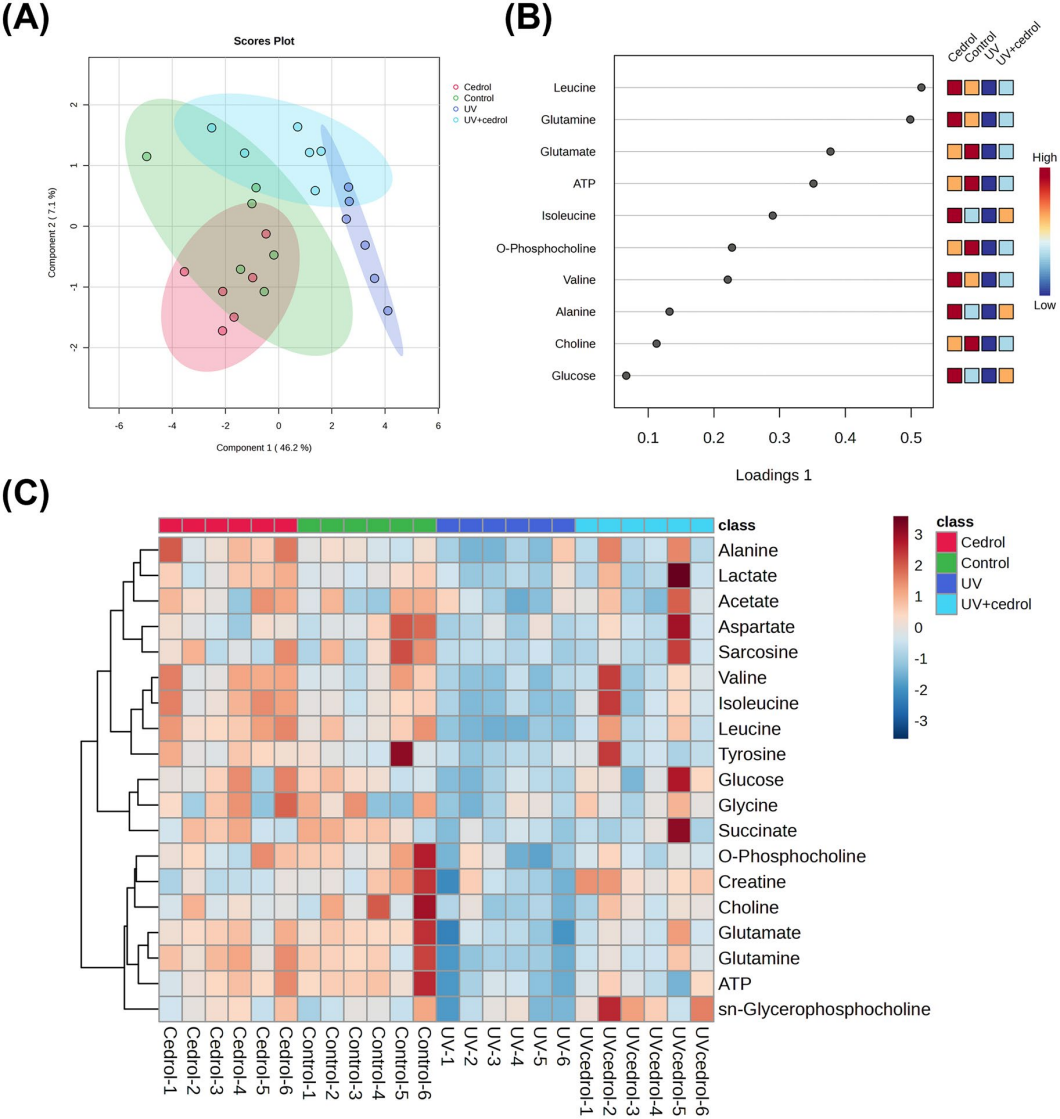
The effects of cedrol on the cell metabolites in UVB-irradiated HaCaT cells. (A) Sparse partial least squares-discriminant analysis (sPLS-DA) analysis of ^1^H NMR spectral data from HaCaT cells. Data are presented as mean ± SD (*n* = 6). The score plots were separated by components 1 (46.2%) and 2 (7.1%) of the total variance in the four groups of cell sample extracts. (B) The loading plot shows the variables selected by the sPLS-DA model for a given component. (C) Heatmap of the metabolic variations from the four groups of cell sample extracts.

To further characterize the global metabolic differences among treatment groups, multivariate statistical analyses were conducted. Principal component analysis (PCA) was first applied to explore data distribution and capture major sources of variance. However, as an unsupervised method, PCA is limited in its ability to separate groups when intergroup variation is subtle and influenced by biological or environmental noise. Consistent with this limitation, PCA did not yield distinct group separation (Supplementary Figure 1). To overcome this, sparse partial least squares discriminant analysis (sPLS-DA) was employed as a supervised method to enhance class discrimination and identify metabolites contributing most to group differences. The optimal model performance (25% error rate) was achieved using four components (Supplementary Figure 2). The resulting sPLS-DA score plot (Figure 7(A)) showed distinct separation between the UVB group and the other groups (control, cedrol, and UVB + cedrol), underscoring the significant metabolic impact of UVB exposure. The UVB + cedrol group clustered closely with the control and cedrol groups, indicating that cedrol treatment restored the metabolic profile toward a non-stressed state. Key metabolites contributing to group discrimination were identified *via* the loading plot (Figure 7(B)). A heatmap visualization (Figure 7(C)) demonstrated that most discriminatory metabolites were downregulated in the UVB group and restored in the UVB + cedrol group, supporting the metabolic restorative effects of cedrol.

**Figure 8.**
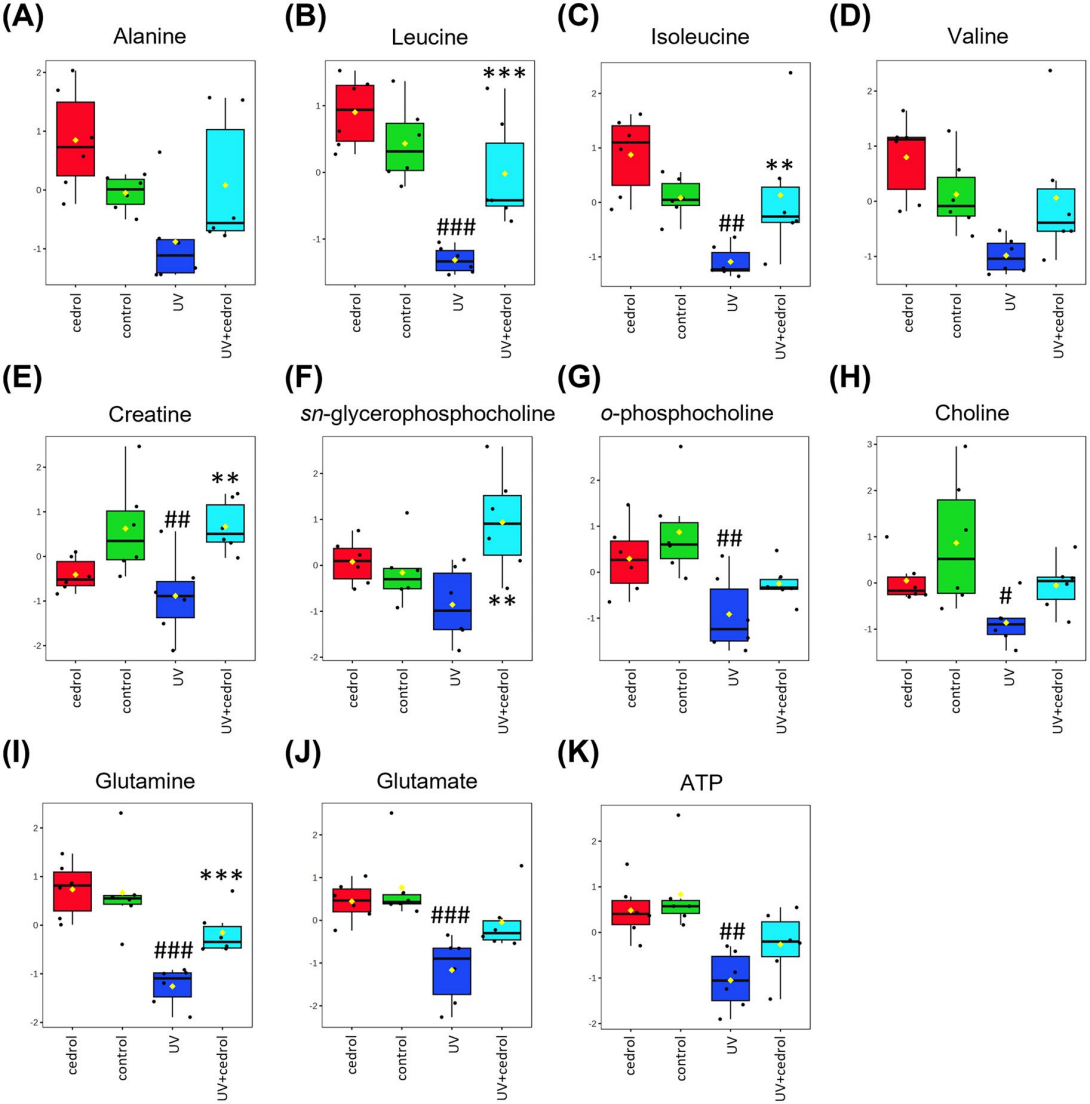
The quantitative levels of significant metabolites detected in HaCaT cell extracts. (A–K) The quantitative levels of significant metabolites detected in HaCaT cell extracts. Data are presented as mean ± SD (*n* = 6). Statistical analysis was performed using one-way ANOVA followed by Tukey’s HSD post hoc test. #*p* < 0.05, ##*p* < 0.01, ###*p* < 0.001 vs. the control group; **p < 0.01, ***p < 0.001 vs. UVB group.

Further quantitative analysis revealed that UVB exposure significantly decreased the levels of several amino acids, including leucine, isoleucine, valine, alanine, glutamine, and glutamate, indicating impairment of amino acid metabolism and potential suppression of protein synthesis (Figure 8). Additionally, ATP and creatine levels were markedly reduced, suggesting disruption of energy metabolism. Metabolites associated with cell membrane integrity and phospholipid turnover, such as *o*-phosphocholine, *sn*-glycero-3-phosphocholine, and choline, were also significantly diminished, indicating that UVB irradiation may compromise membrane stability. Importantly, cedrol treatment significantly restored the levels of many of these metabolites, including glutamate, ATP, and *o*-phosphocholine, to near-control levels, indicating that cedrol mitigates UVB-induced metabolic impairments.

In summary, these metabolomic findings demonstrate that UVB irradiation induces substantial alterations in cellular metabolism, particularly affecting amino acid biosynthesis, energy production, and membrane phospholipid metabolism. Cedrol treatment effectively reversed these metabolic perturbations, suggesting its potential to restore metabolic homeostasis and highlighting its promise as a therapeutic agent for attenuating UVB-induced cellular stress and photoaging.

## Discussion

The skin is composed of three primary layers: the epidermis, dermis, and subcutaneous tissue, with the epidermis serving as the first line of defense. The epidermis comprises multiple layers of keratinocytes that are stratified according to their differentiation status into the basal layer, spinous layer, granular layer, and the outermost stratum corneum (Graham et al. 2019). While the stratum corneum has traditionally been regarded as the principal barrier of the skin, recent studies have highlighted the critical role of tight junctions (TJs) within the epidermis in maintaining barrier function. These structures not only act as physical barriers but also regulate paracellular permeability (Svoboda et al. 2016). Among external environmental factors, ultraviolet (UV) radiation, particularly UVB, is a major contributor to skin barrier disruption. Chronic UVB exposure induces photoaging by promoting the generation of reactive oxygen species (ROS), leading to elevated oxidative stress, mitochondrial dysfunction, metabolic dysregulation, and compromised barrier integrity (Yuki et al. 2011; He et al. 2022). In this study, we demonstrate that cedrol—the predominant constituent of *Cunninghamia lanceolata* var. *konishii* essential oil—reverses HaCaT keratinocytes from UVB-induced cellular damage through multiple mechanisms.

The skin functions as the body’s primary defense against environmental insults. Prolonged exposure to external stressors, particularly ultraviolet (UV) radiation, induces oxidative stress that compromises skin structure and function, thereby accelerating the aging process (Valacchi et al. 2012). Among these factors, UV radiation stimulates the generation of reactive oxygen species (ROS), leading to oxidative damage. Mitochondria, while serving as the primary source of cellular energy, are also major sites of ROS production. Under environmental stress, mitochondrial function is readily impaired, and dysfunctional mitochondria further exacerbate ROS production, creating a self-perpetuating cycle of damage (Birch‐Machin and Bowman 2016). This phenomenon underpins the mitochondrial theory of aging, which posits that excessive ROS, mitochondrial DNA (mtDNA) damage, and mitochondrial dysfunction mutually reinforce one another, progressively driving cellular aging. Chronic UV exposure has been shown to increase mtDNA deletions in skin cells, resulting in reduced ATP production and disrupted cellular energy metabolism and homeostasis (Krishnan et al. 2004). Thus, oxidative stress and mitochondrial dysfunction are key contributors to skin aging, particularly UV-induced photoaging, where mitochondrial damage is closely associated with cellular senescence. Restoring mitochondrial function has therefore emerged as a promising strategy to delay photoaging and preserve skin health (Naidoo et al. 2018). In this study, we demonstrate that cedrol enhances cell viability in a dose-dependent manner and significantly attenuates UVB-induced cellular damage. This restorative effect is accompanied by a notable reduction in intracellular ROS levels, underscoring cedrol’s antioxidant properties, consistent with prior findings (Hu et al. 2024). Furthermore, cedrol restores mitochondrial membrane potential (Δψm) and increases ATP production, indicating enhanced mitochondrial function. Mechanistic analyses revealed that cedrol reverses the UVB-induced downregulation of key regulators of mitochondrial biogenesis and antioxidant defense, including SIRT1, PGC-1α, Nrf2, and TFAM (Alattar et al. 2022). Interestingly, cedrol did not significantly influence AMPK activation, suggesting that its restorative effects may be mediated through AMPK-independent pathways, possibly involving SIRT1 activation.

Tight junction (TJ) proteins are critical for maintaining skin barrier integrity by preventing transepidermal water loss and protecting against pathogen infiltration (Yuki et al. 2011). In this study, we demonstrated that cedrol preserves the expression of key TJ proteins, including ZO-1, occludin, and claudin-3, in UVB-irradiated HaCaT cells, suggesting its potential to stabilize the epidermal barrier and mitigate UVB-induced dysfunction. The skin and gastrointestinal tract represent the body’s two primary interfaces with the external environment. Despite their distinct embryonic origins, these tissues share significant structural and functional features. Both comprise highly specialized epithelial cells that serve as physical barriers, modulate immune responses, and maintain tissue homeostasis (Hu et al. 2018; Kostouros et al. 2020). This resemblance supports the concept of evolutionary parallelism in barrier and immune regulatory mechanisms between the skin and gut (Thye et al. 2022). Our previous research demonstrated that cedrol enhances TJ protein expression and reinforces intestinal epithelial barrier integrity (Xu et al. 2025). Given the structural and regulatory similarities between intestinal and epidermal epithelia, we hypothesize that cedrol may exert comparable restorative effects on the skin barrier. This hypothesis aligns with the emerging concept of the ‘gut–skin axis’, which posits that intestinal barrier integrity can influence systemic health and, by extension, skin homeostasis. Collectively, our findings not only reinforce the barrier-restorative effects of cedrol in the gut but also provide new evidence supporting its efficacy in preserving skin barrier function. These results position cedrol as a promising cross-tissue epithelial modulator with potential therapeutic value for disorders associated with epithelial barrier dysfunction.

Ultraviolet (UV) radiation from sunlight, particularly UVB, is a major external factor affecting skin health. UVB can penetrate the epidermis and reach the dermis (to a depth of approximately 200 μm), where it induces a cascade of molecular and cellular alterations (Bernard et al. 2019). Among the key mediators of skin homeostasis are metabolites-low molecular weight compounds involved in numerous biochemical processes (Elpa et al. 2021). Metabolomic analyses have shown that UVB exposure significantly disrupts cellular metabolism, notably by decreasing levels of several amino acids (e.g., leucine, isoleucine, valine, glutamine, glutamate) and energy-related metabolites (e.g., ATP, creatine), indicating impaired amino acid metabolism and energy production. Previous studies have also demonstrated that UVB-induced senescence in keratinocytes is associated with reduced expression of amino acid transporters, resulting in lower intracellular concentrations of glycine, alanine, and leucine (Bauwens et al. 2023). In addition to disrupting amino acid metabolism, UVB exposure suppresses critical energy-producing pathways in the skin, including glucose utilization, lactate production, glycolysis, the tricarboxylic acid (TCA) cycle, and fatty acid β-oxidation (Hosseini et al. 2018). In this study, we found that cedrol partially reversed these UVB-induced metabolic disturbances, restoring metabolite levels to near those observed in control groups. Amino acids serve not only as structural components of the skin but also contribute to hydration, barrier function, cell proliferation, and tissue repair (Noguchi and Djerassi 2009). In particular, glutamine plays a key role in cellular division and tissue regeneration by serving as both an energy substrate and a nitrogen donor, thereby supporting essential cellular functions and wound healing (Collins 2002). The demand for amino acids increases markedly during skin injury or regeneration. Our results demonstrate that cedrol stabilizes amino acid metabolism and enhances cellular energy production, thereby mitigating UVB-induced metabolic dysfunction. These effects further substantiate its restorative role in cellular defense and skin repair processes.

Several limitations of the present study should be acknowledged. Although UVB was chosen as the primary stressor due to its well-established role in inducing acute epidermal damage and mitochondrial dysfunction, UVA also contributes to skin photoaging, particularly in the deeper dermal layers; future studies could include UVA or combined UV exposure to better reflect physiological conditions. While mitochondrial function and tight junction protein expression were assessed, direct functional assays of barrier integrity (e.g., transepithelial electrical resistance or permeability assays) were not performed, and these should be incorporated in follow-up studies to fully evaluate barrier restoration. As the study was conducted entirely *in vitro* using HaCaT keratinocytes, the physiological relevance and translatability to human skin remain speculative, and *in vivo* investigations are warranted to validate the therapeutic potential of cedrol in a more complex tissue environment. Despite these limitations, the present study provides important insights into the restorative effects of cedrol on UVB-induced cellular damage and supports further investigation of its potential as a post-exposure therapeutic agent.

In summary, this study demonstrates that cedrol effectively reverses UVB-induced oxidative stress, mitochondrial dysfunction, metabolic disturbances, and skin barrier impairment in HaCaT keratinocytes. Its restorative effects appear to be mediated, at least in part, through antioxidant activity and the activation of the SIRT1-PGC-1α-Nrf2-TFAM signaling axis, leading to improved mitochondrial function and restoration of cellular metabolic homeostasis. These findings underscore cedrol’s potential as a promising candidate for preventing and repairing UV-induced skin damage, supporting its application in future skincare and dermatological formulations.

## Conclusions

In conclusion, using a UVB-induced photoaging model in HaCaT cells, this study demonstrated that cedrol activates mitochondrial biogenesis pathways and enhances intracellular ATP production, thereby supplying the energy required to support tight junction protein expression (ZO-1, occludin, and claudin-3) and maintain epidermal barrier integrity. Cedrol also effectively reduces intracellular ROS levels, restores mitochondrial membrane potential, and ameliorates UVB-induced disruptions in amino acid and energy metabolism. These results indicate that cedrol exerts its restorative effects through multiple mechanisms, including antioxidant activity, promotion of mitochondrial function, and stabilization of cellular metabolism, all of which contribute to the restoration of UVB-impaired skin barrier function. Collectively, these findings underscore the potential of cedrol as a natural bioactive ingredient for anti-photoaging applications and support its promising role as a restorative or therapeutic agent in cosmetic and dermatological formulations.

## Supplementary Material

Supplementary.docx

## Data Availability

The data supporting this study’s findings are available in this article’s supplementary materials.
