# Supplementary material for: Cedrol prevents UVB-induced photoaging by restoring mitochondrial function, metabolic homeostasis, and skin barrier integrity in HaCaT cells
Source: Pharm Biol. 2025 Nov 10;63(1):859–76. doi: 10.1080/13880209.2025.2583837 (PMC12608100; doi:10.1080/13880209.2025.2583837)
Supplement: Supplementary.docx [file IPHB_A_2583837_SM9804.docx]

**Cedrol prevents UVB-induced photoaging by restoring mitochondrial function, metabolic homeostasis, and skin barrier integrity in HaCaT cells**

**Mo-Rong Xu^a,b^, Chia-Hsin Lin^c^, Hsun-Hua Lee^d,e,f,g^, Sheng-Yang Wang^a,b,h,i*^**

^a^Doctoral Program in Microbial Genomics, National Chung Hsing University and Academia Sinica, Taichung 402, Taiwan

^b^Department of Forestry, National Chung-Hsing University, Taichung 402, Taiwan

^c^Department of Chinese Pharmaceutical Science and Chinese Medicine Resources, China Medical University, Taichung 404, Taiwan

^d^Department of Neurology, Taipei Medical University Hospital, Taipei Medical University, Taipei 110, Taiwan

^e^Department of Neurology, School of Medicine, College of Medicine, Taipei Medical University, Taipei 110, Taiwan

^f^Dizziness and Balance Disorder Center, Taipei Medical University Hospital, Taipei Medical University, Taipei 110, Taiwan

^g^Department of Neurology, Shuang Ho Hospital, Taipei Medical University, Taipei 110, Taiwan

^h^Program in Specialty Crops and Metabolomics, Academy of Circular Economy, National Chung Hsing University, Nantou 540, Taiwan

^i^Agricultural Biotechnology Research Center, Academia Sinica, Taipei 108, Taiwan

*Correspondence

Sheng-Yang Wang,

Department of Forestry, National Chung-Hsing University, 250 Kuo-Kuang Road, Taichung 402, Taiwan

Fax: +886-4-22873628; Phone: +886-4-22840345 ext. 138.

E-mail: [taiwanfir@dragon.nchu.edu.tw](mailto:taiwanfir@dragon.nchu.edu.tw) (S-Y. Wang)

**
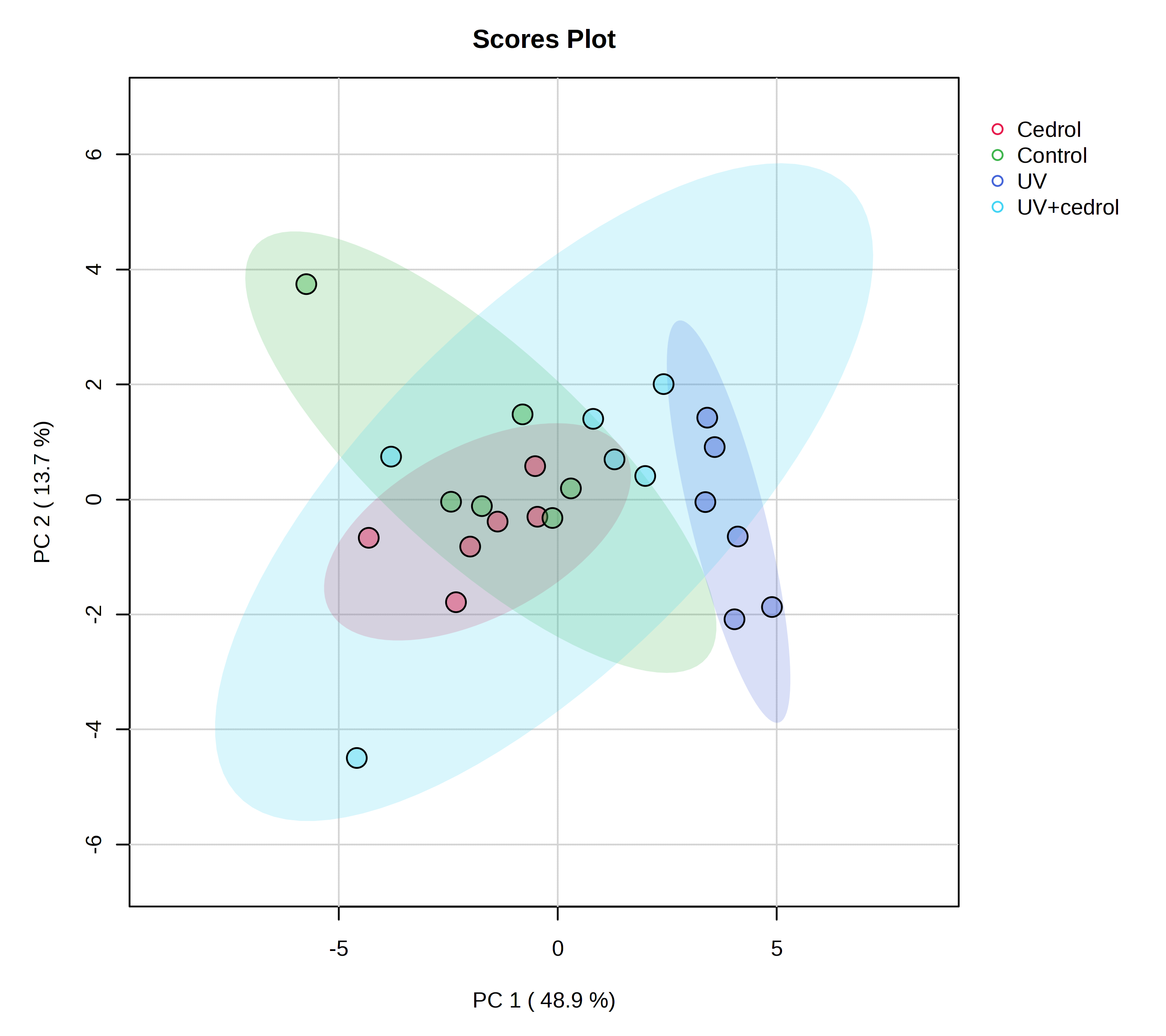
**

Supplementary Figure 1 The scatter plots of Principal components analysis of ^1^H-NMR spectral data from the four groups of HacaT cells.

**
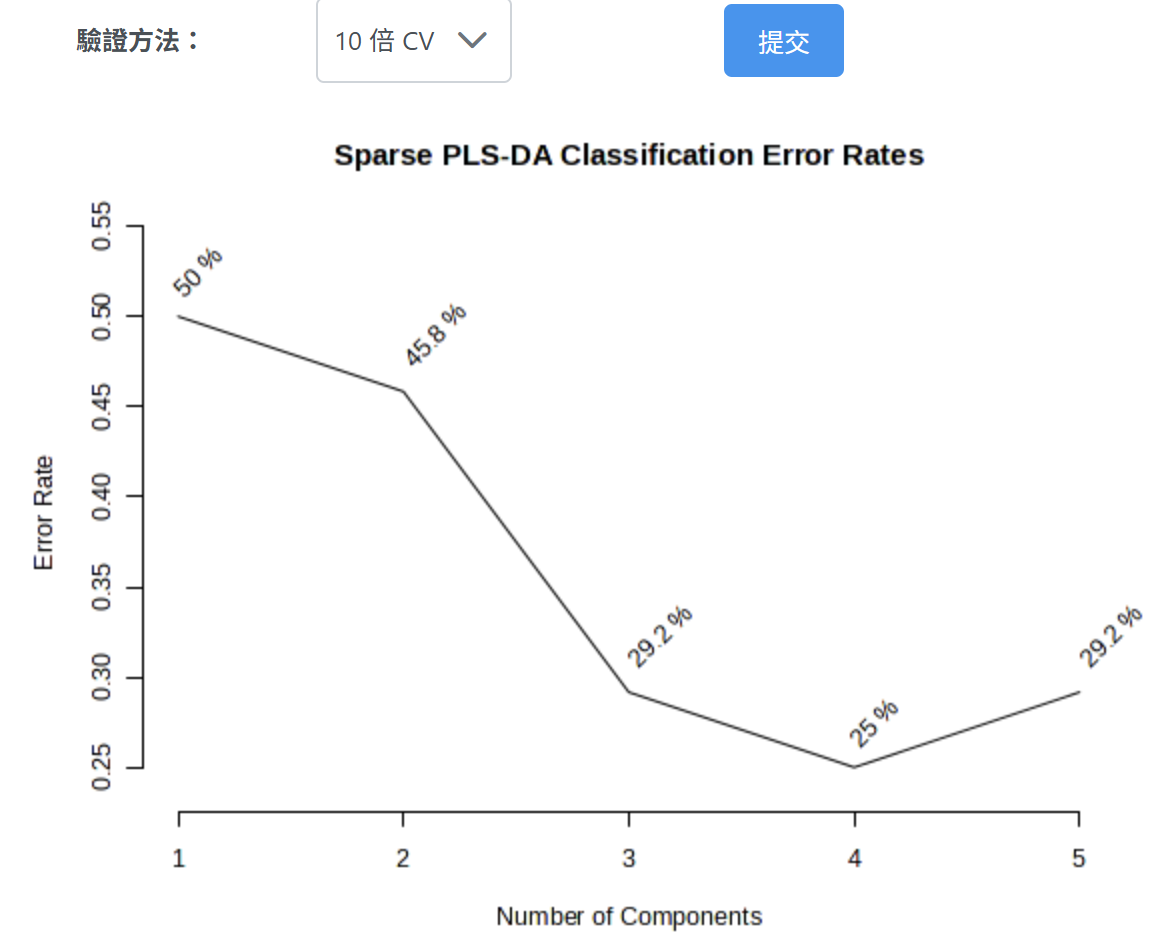
**

Supplementary Figure 2 The error rates of sPLS-DA classification.

Supplementary Table 1. GS-MS analysis of essential oil of *Cunninghamia lanceolate* var. *konishii*.

| **RT** | **Concentration%** | **Constituent** | **Identification** |
| --- | --- | --- | --- |
| 21.16 | 0.70 | *α*-Terpineol | KI/MS/ST |
| 31.12 | 8.66 | *α*-Cedrene | KI/MS/ST |
| 31.44 | 2.46 | *β*-Cedrene | KI/MS/ST |
| 31.91 | 0.38 | *cis*-Thujopsene | KI/MS |
| 34.91 | 0.36 | Cuparene | KI/MS/ST |
| 35.04 | 0.43 | *α*-Chamigrene | KI/MS/ST |
| 38.39 | 1.06 | Globulol | KI/MS |
| 38.97 | 78.48 | Cedrol | KI/MS/ST |
| 39.40 | 1.05 | *epi*-Cedrol | KI/MS/ST |
| 39.75 | 1.59 | *γ*-Eudesmol | KI/MS/ST |
| 40.61 | 2.87 | *α*-Cadinol | KI/MS/ST |

RT: retention tims. KI: Kovats retention index on a DB-5MS column in reference to *n*-alkanes. ST: Authentic standard compounds. MS: NIST and Wiley libraries literature.
